# Confounds in the Data—Comments on “Decoding Brain Representations by Multimodal Learning of Neural Activity and Visual Features”

**DOI:** 10.1109/TPAMI.2021.3121268

**Published:** 2022-11-07

**Authors:** Hamad Ahmed, Ronnie B. Wilbur, Hari M. Bharadwaj, Jeffrey Mark Siskind

**Affiliations:** Elmore Family School of Electrical and Computer Engineering, Purdue University, West Lafayette, IN 47907 USA.; Department of Speech, Language, and Hearing Sciences and the Department of Linguistics, Purdue University, West Lafayette, IN 47907 USA.; Weldon School of Biomedical Engineering and the Department of Speech, Language, and Hearing Sciences, Purdue University, West Lafayette, IN 47907 USA.; Elmore Family School of Electrical and Computer Engineering, Purdue University, West Lafayette, IN 47907 USA.

**Keywords:** Object classification, EEG, human vision, neuroscience, neuroimaging, brain-computer interface

## Abstract

Neuroimaging experiments in general, and EEG experiments in particular, must take care to avoid confounds. A recent TPAMI paper uses data that suffers from a serious previously reported confound. We demonstrate that their new model and analysis methods do not remedy this confound, and therefore that their claims of high accuracy and neuroscience relevance are invalid.

## 1 Introduction

A recent paper [8] presents a novel neural-network architecture, EEGChannelNet, for determining object class from EEG signals recorded from human subjects observing ImageNet [1] images as stimuli. *Inter alia*, it claims:

1. EEGChannelNet can decode object class from EEG signals better than prior work.
2. A training regimen that jointly fine tunes an image classifier while training EEGChannelNet, using a triplet loss that associates both positive and negative image samples with EEG samples, leads to an improved EEG classifier.

Here, we present novel evidence to refute these claims. We note that prior work [6] has already demonstrated other problems, namely:

- The data used in [8] (from Spampinato *et al.* [9]) suffers from a confound (training and test samples coming from the same block with stimuli from a single class) and thus exhibits abnormally high classification accuracy with many different classifiers. When analyzed across subjects to eliminate this confound, accuracy degrades to chance.
- New data collected with a block design also exhibits abnormally high classification accuracy with all of the same classifiers. Accuracy degrades to chance when this new data is bandpass filtered. Likewise, accuracy degrades to chance with new data collected to eliminate the confound: randomized trials and trials where the training and test data have different class presentation order.

Li *et al.* [6] also noted the well-documented slow spectral change in EEG. No amount of filtering can remove the confound.

Here, we document problems with the classifiers and training regimen:

- Their new classifier EEGChannelNet exhibits the same flawed characteristics as the LSTM used in Spampinato *et al.* [9], addressed in [6]. This refutes claim 1.
- Two additional classifiers evaluated by Palazzo *et al.* [8], EEGNet [5] and SyncNet [7], also exhibit the flawed characteristics.
- The joint training regimen exhibits the same flawed characteristics. This refutes claim 2.

All remaining claims [8] are contingent on the confounded data, which results in refutation of the entire paper.

## 2 Method

We attempted to follow the experimental method in [8] and [6] as closely as possible. The appendix in the supplementary material, which can be found on the Computer Society Digital Library at http://doi.ieeecomputersociety.org/10.1109/TPAMI.2021.3121268, available online, presents the details. In all cases, we report the average of accuracy on the validation and test sets after the full training regimen.

## 3 Results

We report below the new results from EEGNet, SyncNet, and EEGChannelNet (abbreviated below as EEGCN) along with the results from Li *et al.* [6].^1^ We first replicate the experiment of Spampinato *et al.* [9] on the block-design data collected by them with their original splits where the test sets come from the same blocks as the training sets.^2^

**Table:** 

Table	subject	LSTM	*k*-NN	SVM	MLP	1D CNN	EEGNet	SyncNet	EEGCN
1		94.7%*	42.2%*	94.4%*	45.8%*	96.7%*	79.2%*	82.8%*	65.0%*
		+Inception v3 [10]		+ResNet-101 [2]		+DenseNet-161 [3]		+AlexNet [4]	
		EEG	image	EEG	image	EEG	image	EEG	image
1		26.4%*	68.4%*	27.7%*	52.2%*	27.4%*	32.2%*	25.8%*	1.5%

The numbers differ somewhat from [9] and [8] as we use a different code base. Nonetheless, the numbers are qualitatively similar in that all classifiers exhibit high EEG classification accuracy. We next replicate the experiment of [9] on the block-design data collected by them with different splits in a leave-one-subject-out cross-validation paradigm. This allows the test sets to come from different blocks than the training sets.

**Table:** 

Table	subject	LSTM	*k*-NN	SVM	MLP	1D CNN	EEGNet	SyncNet	EEGCN
8		2.7%	3.6%*	3.0%*	3.7%*	3.3%*	2.5%	3.8%*	2.6%
		+Inception v3 [10]		+ResNet-101 [2]		+DenseNet-161 [3]		+AlexNet [4]	
		EEG	image	EEG	image	EEG	image	EEG	image
8		2.5%	56.0%*	3.3%*	46.4%*	3.6%*	34.4%*	3.2%*	2.5%

Note that accuracy drops to chance for all classifiers. The remaining tables report analyses done with our own collected data [6]. First, we replicate the experiment of [9] on data collected with a block design on six new subjects.

**Table:** 

Table	subject	LSTM	*k*-NN	SVM	MLP	1D CNN	EEGNet	SyncNet	EEGCN
31	1	67.9%*	100.0%*	100.0%*	21.5%*	82.3%*	58.3%*	77.4%*	93.8%*
32	2	67.3%*	99.8%*	100.0%*	29.1%*	72.3%*	56.8%*	73.6%*	89.9%*
33	3	71.8%*	99.8%*	100.0%*	37.3%*	95.8%*	89.0%*	92.9%*	97.8%*
34	4	72.0%*	99.8%*	100.0%*	36.0%*	89.6%*	83.7%*	78.6%*	95.4%*
35	5	83.8%*	99.0%*	99.9%*	65.3%*	99.5%*	96.8%*	97.6%*	98.5%*
6	6	70.1%*	97.2%*	99.9%*	38.7%*	95.2%*	86.2%*	93.4%*	96.5%*
		+Inception v3 [10]		+ResNet-101 [2]		+DenseNet-161 [3]		+AlexNet [4]	
		EEG	image	EEG	image	EEG	image	EEG	image
31	1	50.3%*	90.3%*	45.8%*	68.4%*	48.7%*	91.1%*	48.3%*	58.5%*
32	2	43.1%*	90.5%*	38.0%*	67.5%*	38.7%*	90.2%*	38.1%*	58.7%*
33	3	70.2%*	91.3%*	66.8%*	69.5%*	67.6%*	90.2%*	67.0%*	60.2%*
34	4	62.5%*	89.8%*	58.0%*	68.6%*	59.4%*	91.6%*	57.0%*	59.6%*
35	5	90.9%*	90.6%*	90.1%*	66.3%*	90.3%*	92.0%*	90.4%*	65.1%*
6	6	65.2%*	89.9%*	62.9%*	70.9%*	62.5%*	91.0%*	62.0%*	56.6%*

Palazzo *et al.* [8], Table 2 bottom and Table 3, claim that EEGChannelNet obtains higher classification accuracy than [9], EEGNet, and SyncNet on that experiment. The above demonstrates that all classifiers can obtain high classification accuracy on data collected with a block design. We collected two runs of block data from subjects 2–5 and three runs of block data from subject 6. Next, we report the data from the second

**Table:** 

Table	subject	LSTM	*k*-NN	SVM	MLP	1D CNN	EEGNet	SyncNet	EEGCN
55	2	70.4%*	98.4%*	100.0%*	42.9%*	98.8%*	92.7%*	92.8%*	94.3%*
56	3	84.7%*	99.2%*	100.0%*	61.4%*	98.5%*	97.8%*	97.6%*	98.0%*
57	4	63.8%*	99.8%*	100.0%*	17.8%*	92.4%*	89.7%*	86.6%*	93.9%*
58	5	76.9%*	99.1%*	100.0%*	49.9%*	95.7%*	87.2%*	95.8%*	96.5%*
13	6	76.4%*	98.0%*	99.9%*	45.7%*	97.5%*	92.4%*	94.5%*	97.3%*
		+Inception v3 [10]		+ResNet-101 [2]		+DenseNet-161 [3]		+AlexNet [4]	
		EEG	image	EEG	image	EEG	image	EEG	image
55	2	75.6%*	90.8%*	74.1%*	73.6%*	76.4%*	90.1%*	71.8%*	54.8%*
56	3	93.2%*	87.8%*	92.4%*	70.1%*	91.8%*	90.9%*	91.9%*	61.5%*
57	4	59.8%*	89.8%*	53.7%*	70.1%*	57.2%*	90.5%*	54.2%*	60.1%*
58	5	82.4%*	91.1%*	80.6%*	70.3%*	81.3%*	91.6%*	78.8%*	59.2%*
13	6	81.2%*	92.0%*	80.0%*	72.8%*	80.5%*	89.7%*	80.9%*	56.4%*

and third

**Table:** 

Table	subject	LSTM	*k*-NN	SVM	MLP	1D CNN	EEGNet	SyncNet	EEGCN
14	6	91.5%*	96.1%*	99.9%*	85.0%*	99.1%*	97.6%*	98.3%*	96.9%*
		+Inception v3 [10]		+ResNet-101 [2]		+DenseNet-161 [3]		+AlexNet [4]	
		EEG	image	EEG	image	EEG	image	EEG	image
14	6	86.8%*	87.3%*	86.3%*	65.1%*	85.7%*	90.6%*	87.3%*	48.7%*

block runs. These concur with the third table above. As discussed in Li *et al.* [6], the analyses in [9] erroneously omitted the bandpass filtering described in that paper. We next repeat the analyses in the above three tables with bandpass filtering added, respectively.

**Table:** 

Table	subject	LSTM	*k*-NN	SVM	MLP	1D CNN	EEGNet	SyncNet	EEGCN
21	1	21.0%*	2.5%	4.1%*	3.2%	62.7%*	33.4%*	28.7%*	4.9%*
22	2	10.4%*	2.7%	3.0%	2.7%	50.7%*	18.0%*	20.9%*	3.3%
23	3	6.0%*	3.0%	3.3%	2.5%	50.4%*	14.8%*	20.7%*	4.1%*
24	4	15.2%*	3.4%	4.8%*	4.8%*	48.1%*	18.4%*	22.8%*	6.3%*
25	5	26.7%*	3.9%*	8.8%*	8.6%*	70.5%*	43.0%*	35.6%*	13.0%*
4	6	16.5%*	2.1%	3.1%	3.3%	37.8%*	13.5%*	15.6%*	5.6%*
		+Inception v3 [10]		+ResNet-101 [2]		+DenseNet-161 [3]		+AlexNet [4]	
		EEG	image	EEG	image	EEG	image	EEG	image
21	1	6.4% *	83.5%*	6.5%*	55.7%*	6.2%*	68.2%*	5.6%*	6.0%*
22	2	5.1%*	83.7%*	4.5%*	61.8%*	5.5%*	66.9%*	4.7%*	6.1%*
23	3	5.1%*	84.8%*	4.8%*	59.4%*	4.8%*	67.5%*	4.0%*	6.8%*
24	4	7.1%*	82.0%*	6.9%*	59.2%*	6.7%*	69.5%*	5.3%*	5.7%*
25	5	6.1%*	84.6%*	5.4%*	56.9%*	6.0%*	66.9%*	4.8%*	5.9%*
4	6	4.9%*	85.5%*	5.8%*	58.9%*	5.0%*	66.3%*	5.0%*	6.3%*

**Table:** 

Table	subject	LSTM	*k*-NN	SVM	MLP	1D CNN	EEGNet	SyncNet	EEGCN
51	2	7.5%*	2.7%	2.8%	2.9%	50.1%*	20.2%*	18.2%*	3.3%
52	3	6.4%*	2.3%	2.2%	3.2%	51.4%*	13.5%*	19.2%*	4.2%*
53	4	16.0%*	2.3%	4.8%*	5.2%*	48.3%*	20.5%*	24.8%*	6.8%*
54	5	35.8%*	3.4%	9.3%*	9.3%*	71.3%*	44.1%*	40.2%*	8.7%*
11	6	7.2%*	2.8%	2.9%	2.9%	31.2%*	7.3%*	9.1%*	3.2%
		+Inception v3 [10]		+ResNet-101 [2]		+DenseNet-161 [3]		+AlexNet [4]	
		EEG	image	EEG	image	EEG	image	EEG	image
51	2	5.0%*	85.9%*	5.1%*	59.4%*	4.1%*	70.7%*	4.7%*	6.1%*
52	3	3.7%*	86.2%*	3.1%	53.9%*	3.7%*	68.5%*	2.8%	6.1%*
53	4	5.0%*	82.9%*	4.4%*	58.7%*	4.5%*	65.6%*	4.9%*	6.0%*
54	5	7.4%*	84.5%*	7.4%*	57.8%*	7.1%*	68.0%*	7.3%*	5.5%*
11	6	4.5%*	85.8%*	4.8%*	60.6%*	4.6%*	69.3%*	4.9%*	6.3%*

**Table:** 

Table	subject	LSTM	*k*-NN	SVM	MLP	1D CNN	EEGNet	SyncNet	EEGCN
12	6	13.1%*	2.8%	3.3%	4.3%*	39.2%*	14.4%*	17.0%*	5.2%*
		+Inception v3 [10]		+ResNet-101 [2]		+DenseNet-161 [3]		+AlexNet [4]	
		EEG	image	EEG	image	EEG	image	EEG	image
12	6	5.4%*	80.6%*	5.3%*	56.7%*	5.6%*	66.5%*	5.3%*	6.8%*

Accuracy drops to chance for all classifiers. We next report analyses performed on data collected with randomized trials both with

**Table:** 

Table	subject	LSTM	*k*-NN	SVM	MLP	1D CNN	EEGNet	SyncNet	EEGCN
26	1	2.1%	2.2%	3.1%	2.6%	2.6%	2.6%	2.5%	1.7%
27	2	2.8%	2.6%	2.5%	2.2%	2.9%	2.6%	2.1%	2.0%
28	3	2.9%	2.2%	2.9%	2.7%	2.2%	2.6%	2.4%	2.3%
29	4	2.5%	2.2%	2.2%	2.4%	2.7%	2.1%	2.5%	2.2%
30	5	2.5%	2.1%	2.8%	3.3%	2.1%	2.5%	2.6%	2.6%
5	6	2.5%	2.5%	2.4%	3.2%	2.3%	2.9%	2.6%	2.8%
		+Inception v3 [10]		+ResNet-101 [2]		+DenseNet-161 [3]		+AlexNet [4]	
		EEG	image	EEG	image	EEG	image	EEG	image
26	1	2.9%	78.3%*	2.1%	51.7%*	2.6%	65.6%*	2.5%	5.7%*
27	2	2.4%	78.6%*	2.0%	55.1%*	2.3%	64.5%*	2.2%	6.7%*
28	3	2.0%	82.3%*	2.8%	56.8%*	2.3%	65.0%*	3.6%*	6.1%*
29	4	2.3%	82.6%*	2.7%	55.8%*	2.1%	66.3%*	2.4%	7.6%*
30	5	2.7%	77.6%*	2.5%	56.9%*	2.2%	66.7%*	2.7%	5.5%*
5	6	2.4%	76.2%*	1.8%	50.1%*	2.8%	67.5%*	2.2%	5.9%*

and without

**Table:** 

Table	subject	LSTM	*k*-NN	SVM	MLP	1D CNN	EEGNet	SyncNet	EEGCN
36	1	0.9%	1.3%	3.3%	1.1%	2.4%	1.2%	2.1%	2.5%
37	2	2.0%	2.1%	3.6%*	1.2%	3.6%*	4.5%*	3.2%	2.5%
38	3	1.2%	1.9%	2.5%	1.3%	3.0%	3.8%*	3.1%	2.3%
39	4	1.6%	1.3%	3.6%*	1.1%	2.2%	2.9%	2.2%	2.0%
40	5	1.3%	2.2%	2.5%	1.5%	2.8%	1.7%	2.1%	2.5%
7	6	1.2%	1.6%	2.9%	1.0%	2.7%	4.6%*	2.5%	2.1%
		+Inception v3 [10]		+ResNet-101 [2]		+DenseNet-161 [3]		+AlexNet [4]	
		EEG	image	EEG	image	EEG	image	EEG	image
36	1	0.8%	70.6%*	0.6%	63.3%*	0.7%	68.1%*	0.6%	9.6%*
37	2	0.9%	76.9%*	1.6%	55.6%*	1.2%	68.1%*	1.7%	6.7%*
38	3	0.6%	74.8%*	1.0%	53.9%*	1.1%	67.0%*	0.7%	6.9%*
39	4	1.4%	76.8%*	1.1%	56.9%*	1.5%	66.2%*	1.0%	6.9%*
40	5	0.9%	76.8%*	0.5%	59.2%*	0.6%	69.1%*	0.4%	9.4%*
7	6	1.2%	76.0%*	1.1%	59.9%*	1.1%	67.4%*	1.1%	7.2%*

bandpass filtering. Note that accuracy is at chance for all classifiers. We next report an analysis on data collected with randomized trials, where the trial labels are replaced with block indices instead of object class, both with

**Table:** 

Table	subject	LSTM	*k*-NN	SVM	MLP	1D CNN	EEGNet	SyncNet	EEGCN
41	1	8.4%*	2.6%	2.5%	2.4%	50.1%*	15.1%*	19.9%*	3.6%*
42	2	5.7%*	1.8%	2.5%	2.9%	52.2%*	8.9%*	20.0%*	3.2%
43	3	16.0%*	2.2%	2.5%	3.3%	54.8%*	15.2%*	28.5%*	3.8%*
44	4	6.3%*	2.3%	2.9%	3.2%	19.2%*	8.4%*	7.9%*	3.4%
45	5	45.2%*	3.0%	10.5%*	10.1%*	84.1%*	70.6%*	52.5%*	15.1%*
9	6	22.7%*	5.9%*	11.3%*	9.0%*	59.6%*	35.7%*	28.5%*	9.8%*
		+Inception v3 [10]		+ResNet-101 [2]		+DenseNet-161 [3]		+AlexNet [4]	
		EEG	image	EEG	image	EEG	image	EEG	image
41	1	3.2%	75.7%*	3.7%*	52.6%*	3.3%	65.6%*	3.8%*	5.4%*
42	2	3.4%	81.0%*	3.5%*	59.6%*	4.0%*	67.6%*	3.5%*	6.3%*
43	3	3.7%*	79.1%*	3.9%*	54.7%*	4.0%*	67.2%*	4.7%*	5.4%*
44	4	5.2%*	80.8%*	5.3%*	59.7%*	5.2%*	67.5%*	4.8%*	7.7%*
45	5	8.6%*	82.2%*	8.1%*	55.4%*	9.4%*	67.5%*	8.1%*	5.8%*
9	6	7.3%*	81.5%*	7.5%*	53.3%*	7.9%*	67.7%*	7.2%*	6.2%*

and without

**Table:** 

Table	subject	LSTM	*k*-NN	SVM	MLP	1D CNN	EEGNet	SyncNet	EEGCN
46	1	78.3%*	99.8%*	100.0%*	39.2%*	99.6%*	98.6%*	97.7%*	98.5%*
47	2	94.6%*	95.4%*	100.0%*	88.3%*	100.0%*	99.6%*	99.9%*	93.8%*
48	3	87.7%*	99.7%*	100.0%*	81.7%*	98.7%*	99.7%*	99.9%*	99.4%*
49	4	90.7%*	94.8%*	99.8%*	78.3%*	99.7%*	99.2%*	99.5%*	79.8%*
50	5	69.7%*	99.7%*	100.0%*	42.9%*	94.5%*	90.0%*	95.2%*	97.2%*
10	6	95.2%*	99.2%*	100.0%*	89.4%*	100.0%*	99.6%*	99.8%*	96.0%*
		+Inception v3 [10]		+ResNet-101 [2]		+DenseNet-161 [3]		+AlexNet [4]	
		EEG	image	EEG	image	EEG	image	EEG	image
46	1	82.1%*	88.1%*	78.3%*	66.8%*	80.2%*	91.2%*	77.4%*	62.2%*
47	2	82.9%*	80.9%*	82.1%*	68.0%*	85.3%*	89.6%*	84.6%*	35.2%*
48	3	97.8%*	85.0%*	97.4%*	64.9%*	97.5%*	91.7%*	97.3%*	59.9%*
49	4	71.2%*	84.8%*	68.7%*	64.8%*	77.3%*	85.8%*	74.6%*	22.3%*
50	5	77.6%*	87.1%*	75.5%*	67.6%*	77.9%*	91.4%*	74.2%*	58.3%*
10	6	91.7%*	89.0%*	91.6%*	66.5%*	93.4%*	89.9%*	93.1%*	47.1%*

bandpass filtering. In other words, all stimuli in the first block are labeled with class 1, even though they reflect different object classes, all stimuli in the second block are labeled with class 2, even though they reflect different object classes, and so forth. Note that classification accuracy is high for all classifiers, without bandpass filtering, suggesting that they are classifying a spurious correlation between the EEG signal and the block, not the stimulus category. This can be unduly high even with bandpass filtering, as is often the case. The remaining tables report cross-block classification. For subjects 2–6, the first and second block runs presented the stimuli in the same order. For subject 6, the third block run presented the stimuli in a different order. First, we report the average results of training on the first block run and testing on the second, and vice versa, both with

**Table:** 

Table	subject	LSTM	*k*-NN	SVM	MLP	1D CNN	EEGNet	SyncNet	EEGCN
63	2	3.2%*	2.6%	2.5%	2.7%	5.3%*	2.9%	4.2%*	2.8%
64	3	2.4%	2.4%	2.4%	2.7%	3.4%*	4.0%*	2.6%	2.4%
65	4	3.6%*	3.7%*	3.2%	2.7%	4.1%*	3.9%*	4.0%*	3.4%*
66	5	2.7%	2.2%	2.0%	2.3%	2.3%	2.6%	1.8%	2.1%
18	6	2.3%	2.5%	2.5%	2.4%	4.0%*	3.7%*	3.4%*	3.0%
		+Inception v3 [10]		+ResNet-101 [2]		+DenseNet-161 [3]		+AlexNet [4]	
		EEG	image	EEG	image	EEG	image	EEG	image
63	2	2.8%	89.5%*	2.7%	66.5%*	2.8%	76.5%*	2.7%	5.8%*
64	3	3.0%	89.3%*	3.0%	64.2%*	2.9%	71.2%*	2.6%	5.4%*
65	4	2.8%	87.3%*	3.1%	62.8%*	3.4%*	77.7%*	3.0%	4.9%*
66	5	1.7%	89.6%*	1.9%	68.8%*	1.9%	78.9%*	1.8%	5.1%*
18	6	2.7%	92.5%*	3.1%	59.4%*	3.1%	76.6%*	3.2%	6.4%*

and without

**Table:** 

Table	subject	LSTM	*k*-NN	SVM	MLP	1D CNN	EEGNet	SyncNet	EEGCN
59	2	25.9%*	22.9%*	26.9%*	6.3%*	6.7%*	1.8%	5.0%*	9.1%*
60	3	6.7%*	8.1%*	8.0%*	5.0%*	4.7%*	2.6%	2.5%	5.9%*
61	4	37.7%*	42.3%*	40.5%*	6.5%*	13.4%*	5.0%*	9.5%*	11.2%*
62	5	3.3%*	2.5%	2.2%	2.9%	3.8%*	1.4%	2.5%	2.8%
15	6	27.9%*	32.9%*	27.7%*	7.0%*	4.2%*	2.1%	1.7%	10.0%*
		+Inception v3 [10]		+ResNet-101 [2]		+DenseNet-161 [3]		+AlexNet [4]	
		EEG	image	EEG	image	EEG	image	EEG	image
59	2	15.1%*	93.4%*	15.9%*	78.5%*	15.0%*	97.0%*	15.5%*	48.9%*
60	3	9.2%*	95.0%*	8.4%*	75.9%*	7.4%*	97.0%*	8.9%*	64.3%*
61	4	20.4%*	97.1%*	21.9%*	76.6%*	19.9%*	95.8%*	19.3%*	56.2%*
62	5	3.1%	93.7%*	3.4%*	75.8%*	3.4%*	96.9%*	4.1%*	61.5%*
15	6	13.5%*	96.9%*	15.1%*	76.2%*	14.4%*	96.1%*	14.4%*	55.1%*

bandpass filtering. These report analyses between different runs with the same stimulus presentation order. Note that classification accuracy with all classifiers is significantly lower than within-block analyses, but can be above chance. Finally, we report the corresponding results for the first and third block runs,

**Table:** 

Table	subject	LSTM	*k*-NN	SVM	MLP	1D CNN	EEGNet	SyncNet	EEGCN
19	6	2.3%	2.8%	2.6%	2.3%	2.2%	2.2%	2.4%	2.6%
		+Inception v3 [10]		+ResNet-101 [2]		+DenseNet-161 [3]		+AlexNet [4]	
					
		EEG	image	EEG	image	EEG	image	EEG	image
19	6	2.7%	92.1%*	2.4%	60.6%*	2.4%	77.7%*	2.7%	6.1%*

**Table:** 

Table	subject	LSTM	*k*-NN	SVM	MLP	1D CNN	EEGNet	SyncNet	EEGCN
16	6	2.9%	6.7%*	3.3%*	2.4%	3.0%	0.9%	2.0%	1.9%
		+Inception v3 [10]		+ResNet-101 [2]		+DenseNet-161 [3]		+AlexNet [4]	
		EEG	image	EEG	image	EEG	image	EEG	image
16	6	2.7%	96.5%*	2.2%	72.7%*	2.2%	96.4%*	1.3%	43.2%*

and for the second and third block runs.

**Table:** 

Table	subject	LSTM	*k*-NN	SVM	MLP	1D CNN	EEGNet	SyncNet	EEGCN
20	6	2.5%	2.1%	2.4%	2.4%	2.8%	2.1%	2.2%	2.6%
		+Inception v3 [10]		+ResNet-101 [2]		+DenseNet-161 [3]		+AlexNet [4]	
		EEG	image	EEG	image	EEG	image	EEG	image
20	6	3.0%	90.0%*	2.5%	69.3%*	2.3%	78.9%*	2.6%	5.3%*

**Table:** 

Table	subject	LSTM	*k*-NN	SVM	MLP	1D CNN	EEGNet	SyncNet	EEGCN
17	6	3.7%*	6.3%*	2.7%	0.8%	0.2%	1.0%	1.2%	2.9%
		+Inception v3 [10]		+ResNet-101 [2]		+DenseNet-161 [3]		+AlexNet [4]	
		EEG	image	EEG	image	EEG	image	EEG	image
17	6	2.3%	94.1%*	2.9%	69.2%*	3.2%	96.8%*	1.9%	48.2%*

These report analyses between different runs with different stimulus presentation order. Note that classification accuracy with all classifiers is at chance. These results demonstrate that there is a confound not only between training and test samples collected in close temporal proximity within the same block, there also is a second confound between samples collected in different runs but with the same temporal offset from the beginning of the run. Collectively these results demonstrate that EEGNet, SyncNet, and EEGChannelNet exhibit exactly the same flawed pattern of behavior as the LSTM model from Spampinato *et al.* [9]. To summarize, the only experiment designs among those considered above that do not suffer from one or both confounds are the ones with randomized trials (the ninth and tenth tables) and cross-block with different stimulus presentation order (the fifteenth through eighteenth tables). EEGChannelNet accuracy is at chance on these. Since all of the analyses in [8] use the same flawed data as in [9], everything that follows from those analyses is suspect.

Palazzo *et al.* [8] compare EEGChannelNet with EEGNet [5] and SyncNet [7] and claim improved accuracy. The tables above demonstrate that any relative performance difference is artifactual as EEGNet and SyncNet exhibit the same characteristics as EEGChannelNet on faulty data. We make no claim about EEGNet or SyncNet themselves or the experiments reported in Lawhern *et al.* [5] and Li *et al.* [7]. Our concerns arise solely for the use of EEGNet or SyncNet as described in [8] for analyzing the flawed data from [9]. It is interesting to note that the tenth table above indicates that EEGNet, along with the SVM and 1D CNN, achieve accuracy slightly above chance on randomized trials.

For joint training, the resulting image classifier always performs above chance, usually highly above chance, but the resulting EEG classifier exhibits the same broad characteristics as all other classifiers, namely high classification accuracy on designs that exhibit a confound (all tables above except the ninth, tenth, and fifteenth through the eighteenth) and chance on designs that do not (the ninth, tenth, and fifteenth through eighteenth tables).

## 4 Conclusion

We demonstrate here that the claims 1 and 2 in Palazzo *et al.* [8] cannot be maintained because they rely on the flawed dataset from Spampinato *et al.* [9]. Further, the classification experiments therein fail when repeated on properly collected data without this confound (the ninth, tenth, and fifteenth through eighteenth tables).

## Supplementary Material

supp1-3121268

## Footnotes

1. All code and raw data that produced these results is available at http://dx.doi.org/10.21227/x2gf-5324.

2. All tables below report results only for image stimuli, 440ms windows, and the full set of channels. The first column gives the corresponding table from [6], some of which are in the supplementary material, available online. The first portion of each table reports results when training an EEG classifier in isolation. The second portion of each table reports results when jointly training EEGChannelNet on EEG together with various image classifiers on the EEG stimuli taken from ImageNet using triplet loss. Starred values indicate above chance (*p* < 0.005) by a binomial cmf.
