# Supplementary material for: Confounds in the Data—Comments on “Decoding Brain Representations by Multimodal Learning of Neural Activity and Visual Features”
Source: IEEE Trans Pattern Anal Mach Intell. Author manuscript; Available in PMC 2022 Dec 3. (PMC9719395; doi:10.1109/TPAMI.2021.3121268)
Supplement: supp1-3121268 [file NIHMS1848505-supplement-supp1-3121268.pdf]

Appendix: Confounds in the data—Comments on "Decoding Brain Representations by Multimodal Learning of Neural Activity and Visual Features"

Hamad Ahmed, Ronnie B. Wilbur, Hari M. Bharadwaj, and Jeffrey Mark Siskind, *Senior Member, IEEE*

1 Method

We attempted to follow the experimental method in Palazzo et al. [3] as closely as possible. We deviated slightly in some respects, for compatibility with Spampinato et al. [4] and Li et al. [2]. For some aspects, we fleshed out the design from the partial specification in Palazzo et al. [3]. We discuss these here, solely to document precisely what we have done. We do not believe that anything substantive turns on these issues.

All data from Spampinato et al. [4] was used without additional filtering. All data from Li et al. [2] was processed twice, once with only 49–51 Hz notch filtering and once with both notch filtering and 14–71 Hz bandpass filtering. For the LSTM, k-NN, SVM, MLP, 1D CNN, EEGNet, SyncNet, and nonconjoined EEGChannelNet, a 40–480 ms window was used. For the conjoined EEGChannelNet, a 20–460 ms window was used.

We reimplemented EEGNet, SyncNet, and EEGChannelNet in PyTorch to be compatible with our overall evaluation framework, from the descriptions in the original papers, referencing the original Theano code for EEGNet and the original TensorFlow code for SyncNet to ensure correctness.

EEGChannelNet incorporates residual blocks that nominally compute $x := F(x)+x$, $F(x)$ being two convolutional layers, each of kernel size 3×3, the stride being 2 for the first layer and 1 for the second. However $x := F(x) + x$ requires the dimensions of $F(x)$ and x to be the same, which is not the case here. In the original ResNet-152 [1], the dimensions of $F(x)$ equal the dimensions of x except on four occasions, where they do $x := F(x)+G(x)$, where G is a 1×1 convolution with a stride of 2 to downsample x and make its dimensions equal to $F(x)$. We do the same here.

All authors are with Purdue University, West Lafayette, IN 47907 USA. H. Ahmed and J. M. Siskind are with the Elmore Family School of Electrical and Computer Engineering. R. B. Wilbur is with the the Department of Speech, Language, and Hearing Sciences and the Department of Linguistics. H. M. Bharadwaj is with the Weldon School of Biomedical Engineering and the Department of Speech, Language, and Hearing Sciences.
E-mail: ahmed90@purdue.edu, wilbur@purdue.edu, hbharadwaj@purdue.edu, qobi@purdue.edu

The kernel size, stride, dilation, and padding values in Palazzo et al. [3, Fig. 2] for the temporal block are inconsistent with the input and output sizes of the last dimension being 440 and 220 respectively. We correct this by using the padding values 16, 32, 64, 128 and 256 instead of the values 16, 31, 62, 124, 248 given in Palazzo et al. [3, Fig. 2].

The kernel size, stride, dilation and padding values specified for the residual block in Palazzo et al. [3, Fig. 2] is inconsistent with the output size. To achieve the specified output size one must set the padding of the first convolutional layer to 0. But this is inconsistent with the need to do $x := F(x) + G(x)$. To allow G to be a 1×1 convolution with a stride of 2 for downsampling, we set the output size to 110 instead of 109. But this, in turn, renders the final output size from the residual block to be 200×4×14 instead of 200×3×12. This necessitates using a 4×5 convolution instead of a 3×3 convolution to convert it to 50×1×10.

EEGNet and SyncNet were trained using the same method as all models in Li et al. [2] were trained: Adam optimizer with default hyperparameters, 100 epochs, and a learning rate of 0.001. EEGChannelNet was taken as the EEG encoder, producing a 1000-element embedding vector. The unmodified image classifiers (from torchvision) were taken as the image encoders, producing a 1000-element embedding vector, without any additional FC layer. The encoders were converted into an classifiers by appending a ReLU and 1000→40 FC layer. EEGChannelNet was trained alone as a classifier by jointly training the EEG encoder and the 1000→40 FC layer from random initialization, with Adam, using the default hyperparameters, 100 epochs, and a learning rate of 0.001. Each epoch contained each EEG sample exactly once, uniformly shuffling between epochs. Conjoined training first trained the EEG and image encoders without the postpended ReLU and 1000→40 FC layers, with Adam, using the default hyperparameters, 100 epochs, and a learning rate of 0.000001. When using DenseNet-161 as the image encoder, the batch size was set to 14 for our data and to 12 for the data from Spampinato et al. [4] to fit into the GPU. The batch size was set to 16 for all other image encoders. For this training, the EEG encoder was randomly initialized and the image encoder was initialized

with pretrained weights, but left unfrozen. Triplet selection was performed during conjoined training so that each epoch contained each EEG sample exactly once, resampling positive and negative image samples and uniformly shuffling between epochs. Each EEG sample was associated with a uniformly sampled ten crop of the associated image as a positive sample and a non-cropped negative image uniformly sampled from all other images. Negative samples were repeatedly drawn up to 10 times to find the first one where the negative compatibility exceeded the positive compatibility. If none were found within 10 tries, the last sample was selected. The 1000→40 FC layer postpended to the EEG encoder was then trained from random initialization, with the EEG encoder frozen, with Adam, using the default hyperparameters, 500 epochs, and a learning rate of 0.00001. Each epoch contained each EEG sample exactly once, uniformly shuffling between epochs. The 1000→40 FC layer postpended to the image encoder was then trained from random initialization, with the image encoder frozen, with Adam, using the default hyperparameters, 500 epochs, and a learning rate of 0.000001. Each epoch contained a single uniformly sampled ten crop of each image exactly once, resampling ten crops and uniformly shuffling between epochs. Validation and test was performed only on non-cropped images. In all cases, we report the average of accuracy on the validation and test sets after the full training regimen.

REFERENCES

[1] K. He, X. Zhang, S. Ren, and J. Sun. Deep residual learning for image recognition. In *Computer Vision and Pattern Recognition*, pages 770–778, 2016.

[2] R. Li, J. S. Johansen, H. Ahmed, T. V. Ilyevsky, R. B. Wilbur, H. M. Bharadwaj, and J. M. Siskind. The perils and pitfalls of block design for EEG classification experiments. *IEEE Transactions on Pattern Analysis and Machine Intelligence*, 2020. doi: 10.1109/TPAMI.2020.2973153.

[3] S. Palazzo, C. Spampinato, I. Kavasidis, D. Giordano, J. Schmidt, and M. Shah. Decoding brain representations by multimodal learning of neural activity and visual features. *IEEE Transactions on Pattern Analysis and Machine Intelligence*, 2020. doi: 10.1109/TPAMI.2020.2995909.

[4] C. Spampinato, S. Palazzo, I. Kavasidis, D. Giordano, N. Souly, and M. Shah. Deep learning human mind for automated visual classification. In *Computer Vision and Pattern Recognition*, pages 6809–6817, 2017.
